# Responsive neurostimulation for patients with refractory mesial temporal lobe epilepsy: A systematic review and meta-analysis

**DOI:** 10.1016/j.ebr.2025.100774

**Published:** 2025-04-22

**Authors:** Eshita Sharma, Beatriz Westphalen Pomianoski, Rabbia Jabbar, Ayesha Ayesha, Yasmin Picanco Silva, Paweł Łajczak, Aisha Rizwan Ahmed, Oguz Kagan Sahin, Mir Wajid Majeed, Mohammed Raake, Walter Fagundes, Giovani Noll

**Affiliations:** a UCLA, Los Angeles, United States of America; b University Nove de Julho, São Paulo, Brazil; c Fatima Memorial Hospital FMHCMD, Lahore, Pakistan; d Shifa College of Medicine, Islamabad, Pakistan; e Healthcare Institution of South Iceland, Selfoss, Iceland; f Medical University of Silesia, Katowice, Poland; g Jinnah Medical and Dental College, Karachi, Pakistan; h Acibadem Mehmet Ali Aydinlar University, Istanbul, Turkey; i Government Medical College Srinagar, India; j Annamalai University, Chennai, India; k Universidade Federal do Espírito Santo, Vitória, Brazil; l Federal University of Rio Grande do Sul, Porto Alegre, Brazil

**Keywords:** Neurostimulation, Refractory epilepsy, Systematic review, Temporal lobe epilepsy, Meta-analysis

## Abstract

Responsive neurostimulation (RNS) is a well-established adjuvant therapy for reducing seizure frequency in adults with medically refractory partial-onset seizures, particularly in individuals who are either not candidates for surgical resection or remain seizure-prone post-surgery. However, its effectiveness in patients with mesial temporal lobe epilepsy (MTLE) remains unclear. This meta-analysis evaluates the efficacy of RNS therapy in individuals with medically refractory MTLE. A systematic search of PubMed, Cochrane, and Scopus databases was conducted to identify eligible studies. Outcomes assessed included mean seizure frequency reduction, responder rate (proportion of patients achieving ≥50 % reduction in seizure frequency), and the proportion of patients achieving seizure freedom within six months of follow-up. Statistical analyses were performed using STATA. Seven observational studies involving 207 patients were included. RNS was associated with a mean seizure frequency reduction of 68.76 % (95 % CI 57.16–80.37 %; I^2^ = 81.68 %), a responder rate of 67.58 % (95 % CI 46.51–88.66 %; I^2^ = 94 %), and seizure freedom within six months in 28.94 % of patients (95 % CI 3.03–54.86 %; I^2^ = 88 %). Moderate to high heterogeneity was observed across the studies. RNS may represent a viable therapeutic option for patients with MTLE, demonstrating substantial reductions in seizure frequency and a notable proportion of patients achieving seizure freedom. Additional studies are needed to confirm these findings and to explore the comparative efficacy and safety of RNS therapy in relation to other treatment options.

## Highlights

• Meta-analysis of 207 patients supports RNS efficacy in refractory MTLE; RNS therapy may reduce seizure frequency in patients with refractory MTLE; Some patients with MTLE achieved seizure freedom within 6 months with RNS.

## 1 Introduction

Mesial temporal lobe epilepsy (MTLE) represents the most prevalent and treatment-resistant subtype of drug-resistant epilepsy [1]. Surgical intervention can effectively treat some patients; however, many are either unsuitable candidates or continue to experience seizures despite surgery [2]. Recently, responsive neurostimulation has emerged and gained attention as an innovative and promising therapy for medically refractory mesial temporal lobe epilepsy [3].

Responsive neurostimulation (RNS), also known as brain-responsive neurostimulation, entails implanting electrodes within the brain's seizure-onset zones. These electrodes continuously monitor brain activity and deliver targeted electrical stimulation upon detecting seizure-like activity, aiming to disrupt or abort seizures [4,5]. Unlike traditional open-loop stimulation methods, which provide constant stimulation irrespective of the brain's activity, this approach offers a more adaptive and personalized treatment strategy [6].

Haneef et al reported DBS lead to greater reduction in seizure frequency than VNS in generalized epilepsy [7]. Kusyk et al reported that RNS in treatment of drug resistant epilepsy decreases seizure reduction rate by 68 %, but has a strong publication bias [8]. Touma et al reported that seizure freedom at last follow-up increased significantly over time for DBS and RNS, whereas a positive trend was observed for VNS in treatment of drug resistant epilepsy [9]. However no meta-analysis of RNS data has been conducted in the context of MTLE. Large-scale clinical trials (e.g., Geller et al. 2017) [5], have established the effectiveness of RNS in MTLE, but variability in individual study outcomes warrants further quantification. This meta-analysis provides a comprehensive pooled analysis to refine estimates of seizure reduction, responder rates, and seizure freedom in medically refractory MTLE patients.

## 2 Methods

This systematic review and meta-analysis adhered to the rigorous methodological standards outlined by the Cochrane Collaboration. To ensure transparency and reproducibility, we followed the Preferred Reporting Items for Systematic Reviews and Meta-Analyses (PRISMA) statement [10]. Additionally, the study protocol was registered in PROSPERO (CRD42024534174), a public repository for systematic review protocols.

### 2.1 Eligibility criteria

Inclusion in this meta-analysis was restricted to studies meeting all the following eligibility criteria: (1) randomized controlled trials (RCTs) or observational studies comprising (2) human individuals with medically refractory MTLE undergoing RNS that (3) reported at least one endpoint of interest (mean seizure frequency reduction, responder rate (proportion of individuals with ≥50 % reduction in seizure frequency), and seizure freedom rate within at least 6 months of follow-up). We excluded case reports and review articles.

### 2.2 Search strategy and screening

A comprehensive search of PubMed, Scopus, Web of Science, Cochrane, and Embase databases was conducted from inception to April 2024. The following search terms were employed: ‘responsive brain stimulation,’ ‘responsive neurostimulation,’ ‘responsive cortical stimulation,’ ‘RNS,’ ‘partial seizures,’ ‘focal seizures,’ ‘partial onset epilepsy,’ ‘partial epilepsy,’ ‘epilepsies, partial/therapy,’ ‘epilepsies, partial/diagnosis,’ ‘refractory,’ and ‘intractable.’ To enhance search sensitivity, MeSH terms and Boolean operators were used to refine the search strategy: (“responsive brain stimulation” OR “responsive neurostimulation” OR “responsive cortical stimulation” OR “RNS”) AND (“partial seizures” OR “focal seizures” OR “partial onset epilepsy” OR “partial epilepsy” OR “epilepsies, partial/therapy” OR “epilepsies, partial/diagnosis”) AND (“refractory” OR “intractable”). The reference lists of all included studies, along with those of relevant systematic reviews and meta-analyses, were manually examined to identify any additional studies that might have been overlooked.

Search results were imported into the systematic review management software Covidence [11]. Duplicate studies were automatically identified and removed. The remaining literature was independently screened for relevance by two authors (A.A. and R.J.) at the title and abstract level. To ensure the accuracy of the screening process, the first reviewer (A.A.) also conducted a secondary duplicate screening. The second reviewer (R.J.) then verified the inclusion of selected studies against the established eligibility criteria. Full-text articles of potentially eligible studies were retrieved and assessed independently by the same two authors (A.A. and R.J.). Any discrepancies that arose during the screening process were solved by a third author (M.W.M.).

### 2.3 Endpoints and subgroup analyses

The endpoints of interest comprised mean seizure frequency reduction (MSR), responder rate (RR), and seizure freedom rate (SF). Responder rate was defined as the proportion of individuals with ≥50 % reduction in seizure frequency. Seizure freedom rate was the overall proportion of patients who were seizure-free for at least 6 months at the last follow-up. Data extraction was performed independently by two authors (A.A. and R.J.). The extracted data included the first author, year of publication, patient sample size, reported outcomes, and type of intervention.

### 2.4 Quality assessment

Two researchers (E.S. and O.S.) independently assessed bias using the Newcastle-Ottawa Scale (NOS) [12]. The NOS measures bias in three areas: participant selection, group comparability, and outcome or exposure assessment. Each study was rated on a scale from 0 to 9, with a score under 6 indicating a significant risk of bias. Publication bias was investigated through funnel-plot analysis of point estimates according to study weights. Leave-one-out sensitivity analysis and Gailbrath plots were performed to assess individual study effects and heterogeneity.

### 2.5 Statistical analysis

Continuous data was presented as mean and standard deviation. When transformation from other dispersion measures was needed, mean and standard deviation was estimated from median and interquartile range using R function estmeansd assuming data with a non-normal distribution. A single proportion analysis with 95 % confidence intervals (CIs) was used to measure the effects in single-arm analysis. To assess heterogeneity, we employed the Cochran Q test and I^2^ statistics, considering p-values less than 0.10 and I^2^ values greater than 50 % to be significant indicators of heterogeneity. Statistical analysis was performed using Stata 18 (StataCorp, College Station, Texas).

## 3 Results

### 3.1 Study selection and characteristics

**Fig. 1:**
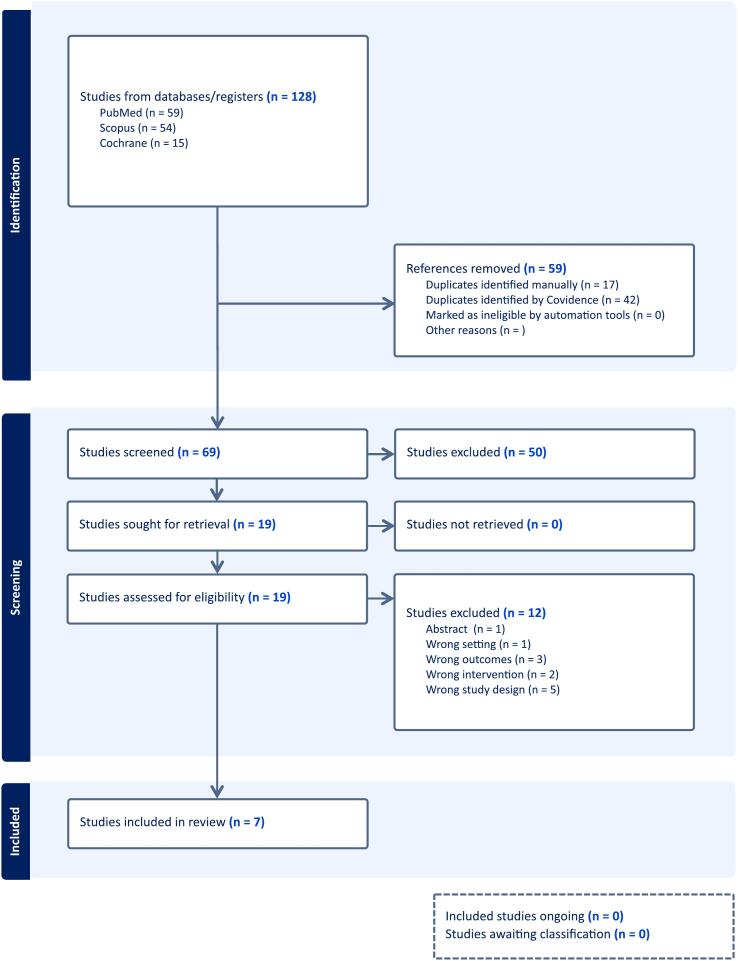
PRISMA flow diagram.

The initial search yielded 128 results. After the removal of duplicate records and ineligible studies, 19 remained and were fully reviewed based on inclusion criteria. Of these, 7 cohort studies [6,7,[13], [14], [15], [16], [17]] were included, comprising 207 patients (Fig. 1). All studies were conducted in hospital settings and included patients from the United States of America. Only one cohort study provided a control group, comparing RNS with vagus nerve stimulation (VNS).(13) Overall, the included patients were 46.3 ± 13.4 years old (mean ± SD), with a baseline duration of epilepsy of 17.5 ± 13.9 years (mean ± SD) and a mean frequency of 19.7 ± 112.2 seizures/month (mean ± SD). Most patients had bilateral MTLE (46.2 % to 100 %), as summarized in Table 1.

**Table 1:** Baseline characteristics of the included studies.

Study	Design	Sample size	Age† (years)	Female gender: % (n)	Epilepsy duration† (years)	Baseline monthly seizure frequency†	Epilepsy side: % (n)	Follow-up, months†
Geller 2017	Observational	111	37.3 ± 11.3	48 % (53)	19.8 ± 12.7	15.1 ± 25.0	Unilateral MTL seizure onsets: 28 % (31) Bilateral MTL seizure onsets: 72 % (80)	73.2 ± 26.4
Hirsch 2020	Observational	Unilateral MTLE: 9	45.1 ± 11.6	N/A	19.5 ± 12.6	N/A	Unilateral: 100 %	26 ± 16.9
		Bilateral MTLE: 15	36.2 ± 13.7	N/A	16.6 ± 15.1	N/A	Bilateral: 100 %	32.3 ± 31.7
Nunna 2020	Observational	10	44.6 ± 7.1	30 % (3)	23.3 ± 12.5	N/A	Bilateral: 100 %	50.4 ± 39.6
Razavi 2020	Observational	150	41.6 ± 16.6	51 % (77)	22.3 ± 11.6	7.7 ± 257	Unilateral: 29 % Bilateral: 71 %	27.6 ± N/A
Wang 2020	Observational	12	39.2 ± 12.1	33.3 % (4)	12.8 ± 9.4	36.3 ± 54.7	Unilateral: 25 % (3) Bilateral: 75 % (9)	43.6 ± 39.1
Ho 2022	Observational	13	38.3 ± 10.4	61.5 % (8)	25.2 ± 12.9	N/A	Unilateral: 53.8 % (7) Bilateral: 46.2 % (6)	57.3 ± 13.7
Charlebois 2022	Observational	22	41.6 ± 11.3	54.5 % (12)	18.0 ± 10.8	N/A	N/A	N/A

N/A = Not available.

† Mean ± SD.

### 3.2 Pooled analysis of all studies

**Fig. 2:**
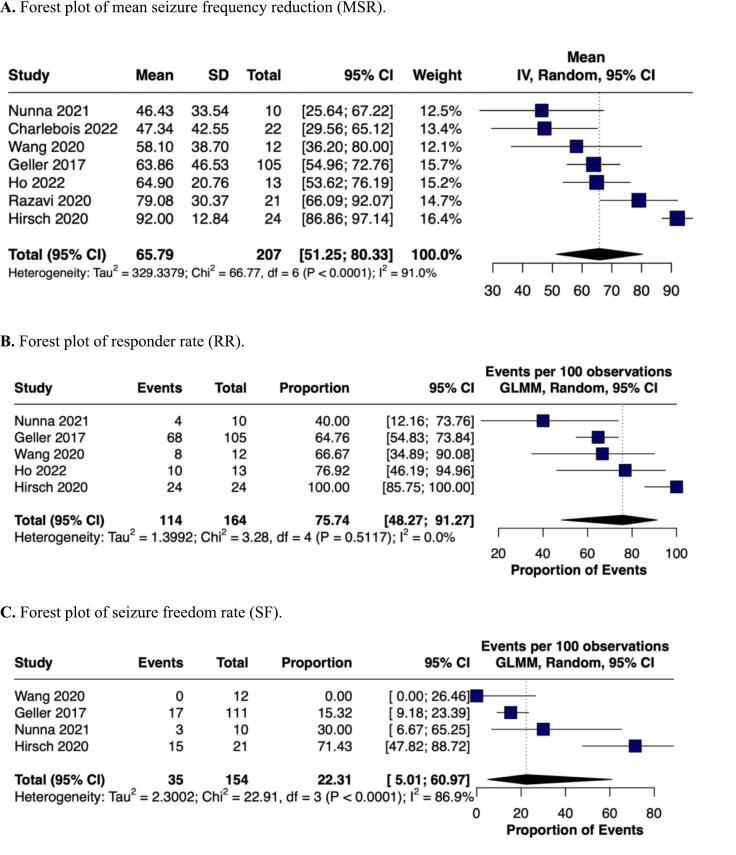
Forest plots for (A) MSR, (B) RR; and (C) SF.

RNS therapy was associated with a significant reduction in the incidence of seizures in 65.79 % (95 % CI 51.25–80.33 %; I^2^ = 91 %) of patients. High heterogeneity was probably related to variable lengths of treatment, follow-up periods, and prior treatment modalities (Table 1). An analysis comprising 164 patients demonstrated that 75.74 % (95 % CI 48.27–91.27 %; I2 = 0 %) of patients achieved the responder rate, with zero heterogeneity among the studies (Fig. 2). Furthermore, another analysis of 4 studies [6,13,14,15] including 154 patients showed that 22.31 % (95 % CI 5.01 %–60.97 %; I^2^ = 88.9 %) of patients achieved seizure freedom at 6 months of follow-up, also with high heterogeneity across the studies (Fig. 2).

### 3.3 Sensitivity analysis

**Fig. 3:**
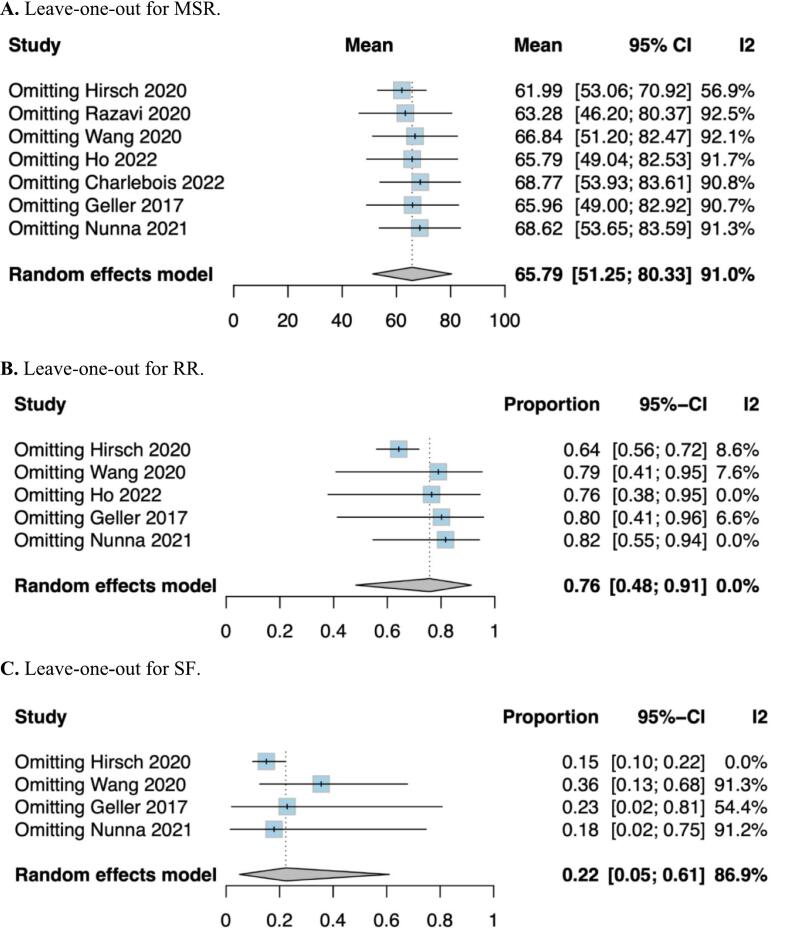
Leave-one-out sensitivity analysis for (A) MSR; (B) RR; and (C) SF.

As shown in Fig. 3, the leave-one-out sensitivity analysis demonstrated that the exclusion of individual studies significantly influenced the overall reduction in mean seizure frequency and responder rate, indicating a high degree of sensitivity in the analysis. For seizure freedom, the analysis revealed that excluding Hirsch et al. [13] (p = 0.00), Wang et al. [15] (p = 0.024), and Geller et al.(6) (p = 0.049) had a substantial impact on the results. Hirsch et al. [13] reported notably higher reductions in seizure frequency, responder rates, and seizure freedom rates compared to other studies. This discrepancy, combined with its small sample size, may suggest potential bias in patient selection or outcome reporting. Conversely, Wang et al. [15] showed a comparatively lower seizure freedom rate, which may be attributable to the limited sample size and scarce number of events, leading to imprecise estimates. Geller et al. [6], one of the studies with the largest sample size and narrower confidence intervals for effect size estimates, exerted a significant influence on both the meta-analysis and the sensitivity analysis results. Additionally, variability in intervention protocols across the included studies likely contributed to differences in outcomes and the observed heterogeneity.

### 3.4 Quality assessment

Overall, the studies reviewed exhibited significant concerns regarding bias across multiple domains. Five studies [7,13,14,16,17] (Ho et al, Charlebois et al, Nunna et al, Razavi et al, and Hirsch et al) demonstrated a high risk of bias concerning the selection of non-exposed cohorts. Three studies [7,13,14] (Nunna et al, Razazvi et al, Hirsch et al) showed either unclear or high risk of bias regarding outcomes not present at the beginning of the study. Four studies [6,7,13,14] (Nunna et al, Razazvi et al, Hirsch et al, Geller et al) exhibited unclear or high risk of bias concerning comparability. Two studies [6,14] (Nunna et al and Geller et al) were identified as having a high risk of bias regarding the adequacy of follow-up. In conclusion, the cohort studies analyzed display notable limitations and biases across various methodological aspects. Individual appraisal of studies is reported in Supplementary Table 1.

**Fig. 4:**
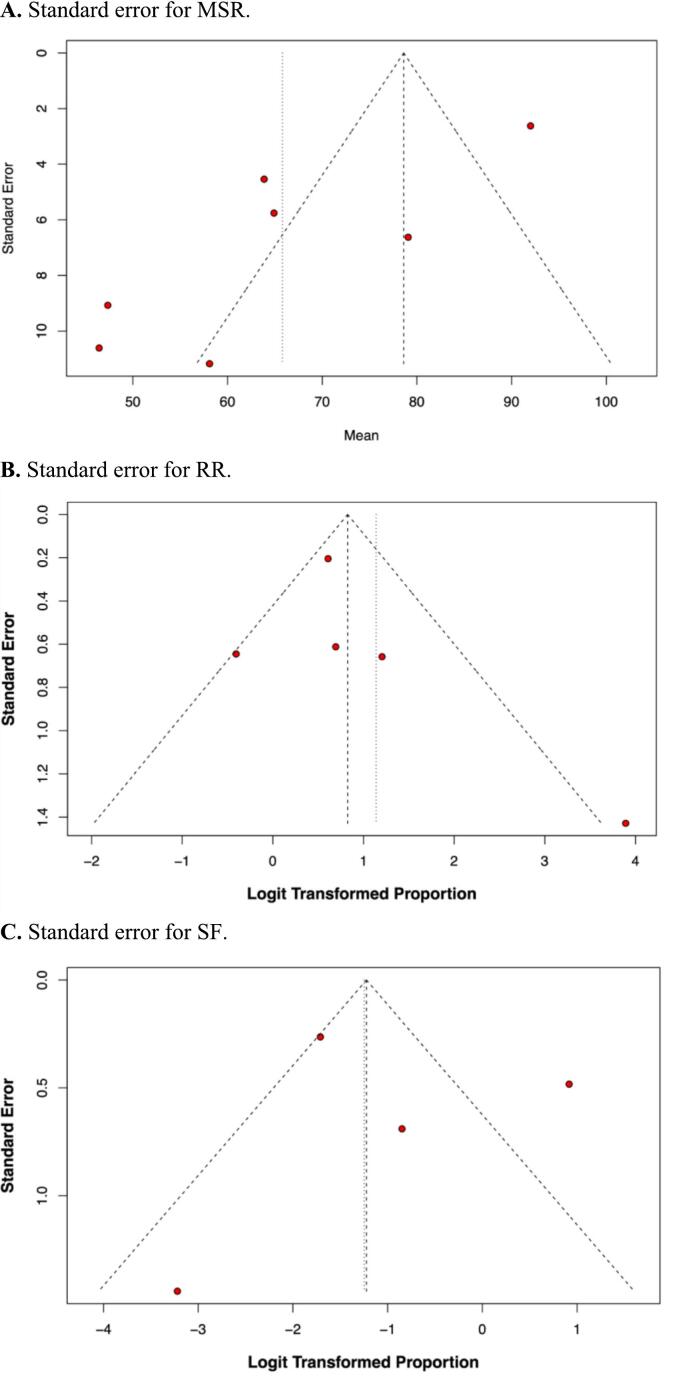
Standard error for (A) MSR; (B) RR; and (C) SF.

Additionally, the symmetry and configuration of the funnel plots for all three outcomes, as illustrated in Fig. 4, suggest that publication bias was not a particular issue in our meta-analysis.

## 4 Discussion

This systematic review and meta-analysis, encompassing seven cohort studies with a total of 207 patients, evaluated the efficacy of responsive neurostimulation (RNS) for seizure control in individuals with drug resistant mesial temporal lobe epilepsy (MTLE). The primary findings demonstrated: (1) an approximately 70 % reduction in mean seizure frequency, (2) a responder rate of nearly 70 %, and (3) seizure freedom achieved by almost 30 % of patients for the minimum of six months from the last follow-up across studies. These outcomes are notable, particularly given the treatment-refractory nature of MTLE.

RNS has shown considerable potential for reducing seizure frequency, achieving higher responder rates, and facilitating seizure freedom in patients with MTLE. For example, one clinical trial included in this analysis [6] randomized 111 patients with MTLE to active or sham stimulation in addition to ongoing anti-seizure medications. In the active stimulation group, seizure frequency reduction, responder rate, and seizure freedom rates were approximately 66 %, 64 %, and 14 %, respectively. Similarly, a 12-week blinded and 84-week open-label randomized controlled trial (RCT) involving 191 patients with medically intractable partial epilepsy reported a 37 % reduction in mean seizure frequency compared to the sham control group [18].

The observed variability in seizure frequency reduction, responder rates, and seizure freedom across studies is likely influenced by both the acute seizure-terminating and chronic neuromodulatory effects of RNS. These outcomes are affected by stimulation parameters, including charge density, which is often empirically increased to enhance therapeutic response [[19], [20], [21]]. However, a direct correlation between charge density and seizure frequency reduction remains unconfirmed.

VTA (volume of tissue activated) depends upon stimulation parameters, RNS cortical strip and depth leads location, and electrode contact per lead [17]. Depth lead is usually longitudinal (trans occipital) where the electrode traverses the long axis of the hippocampus [16], other approaches are orthogonal (lateral) where the electrode is perpendicular to the long axis of hippocampus [16]. Another is the hippocampus sparing approach, used in bilateral MTLE, where the electrode traverses longitudinal axis of extra hippocampal white matter using an occipito-temporal approach [14]. While cortex strip leads are placed sub temporally [13]. If at least two of four electrode contacts of depth leads are in the hippocampus, it is categorized as within the hippocampus, and if more than two of electrode contacts lie outside the hippocampus, then considered as outside hippocampus [6].

Furthermore, differences in follow-up duration across studies may have impacted the results, contributing to heterogeneity in the analyzed endpoints. The efficacy of RNS in modulating seizure activity may also be state-dependent. Stimulation parameters that are effective in one brain state may be less effective or even counterproductive in another, potentially due to state-specific disruptions of pathological cortical synchrony [21]. This highlights the complex interplay between brain state, stimulation parameters, and seizure outcomes.

This meta-analysis represents the first attempt to specifically evaluate the effectiveness of RNS in patients with MTLE. Previous meta-analyses have primarily focused on the use of RNS in generalized and focal drug-resistant epilepsy rather than MTLE specifically. For example, a meta-analysis of non randomized studies in focal epilepsy reported that RNS demonstrated seizure reduction efficacy comparable to deep brain stimulation (DBS), with both modalities outperforming vagus nerve stimulation (VNS) during the first year post-implantation. However, these differences diminished over longer-term follow-up [22]. Similarly, in the pediatric population, another meta-analysis reported a median seizure reduction of 75 %, with 80 % of patients identified as responders [23].

### 4.1 Limitations of study and future directions

This study has important limitations. First, all included studies were observational, with notable variability in the duration of treatment, stimulation thresholds, and follow-up periods. These differences likely influenced the effects of RNS therapy and contributed to the observed heterogeneity. To further investigate this heterogeneity, a leave-one-out sensitivity analysis, and a Galbraith plot were performed, identifying three studies [6,13,15] as having a significant influence on the findings across all three outcomes. However, due to the limited number of included studies, small sample sizes, and variability in reported endpoints, subgroup analyses or meta-regressions were not feasible.

Another limitation is the lack of comparative studies evaluating the efficacy of RNS against other neuromodulatory treatments for drug-resistant epilepsy (DRE), such as VNS and DBS. Comparative analyses specifically RCT and cohort studies focusing on MTLE are necessary to clarify the relative effectiveness of these approaches.

Future research is warranted to compare RNS specifically in the context of MTLE with other surgical options, such as temporal lobectomy, laser interstitial thermal therapy (LITT), and deep brain stimulation (DBS). Efforts should also focus on the personalization of RNS therapy by optimizing stimulation parameters and duration based on brain state and individual clinical characteristics. Furthermore, additional studies are needed to elucidate the neurobiological mechanisms underlying RNS and to assess its safety and efficacy in pediatric populations.

## 5 Conclusion

This systematic review and *meta*-analysis encompassing 207 patients suggests that RNS therapy might help reduce seizure frequency and achieve seizure freedom for at least 6 months in patients with medically refractory mesial temporal lobe epilepsy (MTLE). However, the limitations inherent to a single-arm meta-analysis design demand further investigation. Well-designed studies with control groups, larger sample sizes, and extended follow-up periods are warranted to further assess the effectiveness of RNS in these patients.

## Availability of data and materials

This manuscript did not generate any new data. All data is available in respective papers.

## Use of artificial intelligence

Gemini AI (Google Inc) artificial intelligence software was used for grammar improvement of parts of this manuscript.

## Clinical trial number

Not applicable.

## Ethical approval

Not applicable for this review.

## CRediT authorship contribution statement

**Eshita Sharma:** Writing – review & editing, Writing – original draft, Visualization, Validation, Supervision, Software, Resources, Project administration, Methodology, Investigation, Funding acquisition, Formal analysis, Data curation, Conceptualization. **Beatriz Westphalen Pomianoski:** Writing – original draft, Investigation, Data curation. **Rabbia Jabbar:** Methodology, Investigation, Formal analysis, Data curation. **Ayesha Ayesha:** Methodology, Formal analysis, Data curation. **Yasmin Picanco Silva:** Writing – review & editing, Visualization, Validation, Supervision, Resources, Project administration. **Paweł Łajczak:** Writing – original draft, Validation, Software, Methodology, Investigation, Formal analysis, Data curation. **Aisha Rizwan Ahmed:** Methodology, Formal analysis, Writing – original draft, Validation. **Oguz Kagan Sahin:** Methodology, Investigation, Formal analysis, Data curation. **Mir Wajid Majeed:** Investigation. **Mohammed Raake:** Writing – original draft, Investigation, Data curation, Conceptualization. **Walter Fagundes:** Writing – review & editing, Visualization, Validation, Supervision, Methodology, Formal analysis. **Giovani Noll:** Writing – review & editing, Visualization, Validation, Methodology, Formal analysis.

## Funding

This paper has received no funding.

## Declaration of competing interest

The authors declare that they have no known competing financial interests or personal relationships that could have appeared to influence the work reported in this paper.
