# Supplementary material for: Responsive neurostimulation for patients with refractory mesial temporal lobe epilepsy: A systematic review and meta-analysis
Source: Epilepsy Behav Rep. 2025 Apr 22;30:100774. doi: 10.1016/j.ebr.2025.100774 (PMC12118465; doi:10.1016/j.ebr.2025.100774)
Supplement: Supplementary Data 1 [file mmc1.docx]

**Supplementary Table 1.** Individual bias assessment of studies according to the Newcastle-Ottawa Scale.

|  | Selection | | | | Comparability | Outcome | | |  |
| --- | --- | --- | --- | --- | --- | --- | --- | --- | --- |
| Study (Year) | Representativeness of Exposed Cohort | Selection of Non-Exposed Cohort | Ascertainment of Exposure | Outcome Not Present at Start | Comparability of cohorts | Assessment of Outcome | Follow-up Length | Adequacy of Follow-Up | Overall |
| Ho 2022 | * | - | * | * | ** | * | * | * | 8 |
| Charlebois 2022 | * | - | * | * | ** | * | * | * | 8 |
| Nunna 2021 | * | - | * | = | - | * | * | - | 4 |
| Razavi 2020 | * | - | * | * | = | * | * | * | 6 |
| Hirsch 2020 | * | - | * | * | - | * | * | * | 6 |
| Wang 2020 | * | * | * | * | ** | * | * | * | 9 |
| Geller 2017 | * | * | * | * | = | * | * | - | 6 |

“=” symbol is used for could not be determined. “-“ symbol is used for no stars given.
